# Genetic Variation and Population Structure of *Clonorchis sinensis*: An In Silico Analysis

**DOI:** 10.3390/pathogens13110991

**Published:** 2024-11-13

**Authors:** Xinhui Zhang, Zhuo Lan, Wei Wei, Aihui Zhang, Hongyu Qiu, Junfeng Gao, Chunren Wang

**Affiliations:** 1 Key Laboratory of Bovine Disease Control in Northeast China, Ministry of Agriculture and Rural Affairs, College of Animal Science and Veterinary Medicine, Heilongjiang Bayi Agricultural University, Daqing 163319, China; zzzhangxinhui@163.com (X.Z.); lannzhuo@163.com (Z.L.); weiweixs213149@163.com (W.W.); zhangaihui2090@163.com (A.Z.); qiuhongyu95@163.com (H.Q.); gaojunfeng_2005@163.com (J.G.); 2 Heilongjiang Province Cultivating Collaborative Innovation Center for the Beidahuang Modern Agricultural Industry Technology, Daqing 163319, China

**Keywords:** *Clonorchis sinensis*, COX1, ITS1, ITS2, genetic variability, phylogenetic analysis

## Abstract

*Clonorchis sinensis* is an important zoonotic parasite that is mainly prevalent in China, Korea, Vietnam and the Russian Far East. To explore the genetic variation and population structure of *C. sinensis,* an in silico analysis was conducted based on mitochondrial cytochrome *c* oxidase subunit 1 (COX1), ribosomal internal transcribed spacer 1 (ITS1) and ribosomal internal transcribed spacer 2 (ITS2) sequences. The sequences obtained from NCBI were truncated for further analyses, including haplotype network, phylogenetic, gene flow, diversity and neutrality analyses. The results showed that there were 20, 11 and 4 haplotypes for COX1, ITS1 and ITS2, respectively. The results of both the haplotype network and phylogenetic analyses indicated that the haplotypes for each type of sequence from the same country were not all clustered together. Haplotype diversity values were all lower than 0.5. Values of nucleotide diversity were higher than 0.005, except for ITS2. Tajima’s D and Fu’s Fs values were all negative, and *p*-values showed significant differences, indicating that the population of *C. sinensis* is growing. Fst values were all lower than 0.05. In conclusion, this study found that there are specific variations of *C. sinensis* in different countries, and the population of this parasite is growing with less genetic variation. The findings provide a crucial foundation for understanding the molecular epidemiology and population dynamics of *C. sinensis*.

## 1. Introduction

*Clonorchis sinensis* is a vital fish-borne zoonotic parasite that parasitizes the liver and bile duct of humans and other animals. Not only does it have a serious impact on animal health and livestock production, but it also poses an enormous threat to human health and public health security. *C. sinensis* is mainly prevalent in China, Korea, Vietnam and the Russian Far East, with about 15 million people infected to date [1]. However, with the growth of international markets, improved transportation systems and increased population mobility, cases have also been reported in non-endemic areas and developed countries, such as Ghana, the United States and Canada [2,3,4]. Moreover, the prevalent species of this parasite in different regions can exchange due to factors such as trade interflow, and this will contribute to the possibility of hybrid species. It is notable that different haplotypes of this parasite are present in several countries [5]. Indicators such as genetic, nucleotide and phylogenetic diversity can provide insight into the fitness, evolutionary potential and history of different species in the environment [6]. They are powerful tools for revealing invasive processes, such as migration, gene flow, genetic drift, genetic variability and population expansion [7]. Thus, understanding the population structure and genetic variability of *C. sinensis* is helpful for further analysis of its genetic evolution and global molecular epidemiology.

At present, mitochondria and ribosomes are very important tools that are often used to analyze population structure and genetic diversity. As one of the most common genes examined for population structure and dynamics, mitochondrial cytochrome *c* oxidase subunit 1 (COX1) is widely used in species identification, phylogenetic relationship and genetic diversity analyses [8]. The COX1 gene of *C. sinensis* from Russia and Vietnam has been analyzed, showing low nucleotide and high haplotype differentiation within and between these two regions; furthermore, there was an explicit geographical vector for the distribution of genetic diversity patterns among the studied populations [9,10]. Ribosomal internal transcribed spacer 1 (ITS1) and internal transcribed spacer 2 (ITS2) have shown high divergence in genetic structure and interspecies relationship studies [9,11,12]. A study reported that the ITS1 gene sequence of *C. sinensis* in Russia and Vietnam played an important role in the genetic evolution of their population [9]. Lee et al. analyzed 18S, ITS1, ITS2 and COX1 sequences of *C. sinensis* and found that the Korean and two Chinese isolates were similar at the DNA sequence level [13]. Thus far, many COX1, ITS1 and ITS2 sequences of *C. sinensis* in different countries have been uploaded to the NCBI database that can be used for in silico analysis.

The purpose of this study was to use a data repository in the public domain, NCBI, to conduct an in silico analysis of *C. sinensis* based on COX1, ITS1 and ITS2 sequences from different regions, which would provide a better understanding of the molecular epidemiology and population structure of *C. sinensis* globally.

## 2. Materials and Methods

### 2.1. Sequence Collection

The sequences of *C. sinensis* used in this study were downloaded from NCBI, which is a USA database, at https://www.ncbi.nlm.nih.gov/ accessed on 20 March 2024. All uploaded COX1, ITS1 and ITS2 sequences were collected from the earliest publication date until 20 March 2024. “COX1”, “ITS1” or “ITS2” and “*C. sinensis*” were used as the keywords. To ensure the precision of the data, sequences were excluded if they were not compatible with the aim of the study. For example, some sequences of other parasites were shown in the results and were excluded. In total, 534 eligible sequences of *C. sinensis* were obtained, including 168 COX1 sequences from 4 countries, 275 ITS1 sequences from 5 countries and 91 ITS2 sequences from 5 countries. Detailed information about these sequences is provided in Table S1.

### 2.2. Alignment and Haplotype Network

The various categories of the downloaded sequences were assembled using Editseq software (7.1) so that each type of sequence was in one file for subsequent operations. Each type of sequence was truncated and kept at the same length, and the file was later saved separately in FASTA format using Molecular Evolutionary Genetics Analysis Version 11 (MEGA 11). DnaSP6 software (6.12.03) was utilized to group sequences from different countries, resulting in the generation and storage of haplotype files in arp and hap formats [14]. The notepad++ tool was utilized to convert the aforementioned file formats into Nexus format to facilitate subsequent operation [15]. We then created a template file and added haplotype and frequency distribution data to the relevant positions of the data module and the corresponding location of traits, respectively. Finally, PopArt (Population Analysis with Reticulate Trees) software (1.7) was leveraged to construct a haplotype network diagram. The configuration of properties, such as character colors, label fonts and illustration fonts, was set via the Edit menu.

### 2.3. Phylogenetic Analyses

Phylogenetic analyses were performed based on the haplotype data of each type of sequence via MEGA 11. As the Maximum Likelihood (ML) method offers high accuracy and authenticity, three phylogenetic trees of different sequences were constructed with this method using the available haplotype sequences of COX1, ITS1 and ITS2. The parameters were set in “PHYLOGENY”, and 1000 bootstrap replicates were used to obtain statistical support for branch specificity. The gene sequences of *Schistosoma japonicum* were used as an outgroup. The phylogenetic trees of the three types of sequences were saved as PDF files. The haplotype colors on different branches were adjusted using Word Processing System Office (WPS), and the distribution of the haplotypes in diverse countries were distinguished according to the colors.

### 2.4. Gene Flow, Diversity and Neutrality Analyses

Haplotype diversity (Hd), nucleotide diversity (π), Tajima’s D, Fu’s Fs, FLD, FLF, Fst and other values were calculated using DnaSP6. Each of the three types of processed sequences were uploaded to DnaSP6 software, and the Hd, π and number of mutations were calculated from “DNA Polymorphism” using the “Analysis” option. The value of parsimony informative sites was obtained via “Polymorphic Sites”. Tajima’s D value was analyzed using “Tajima’s Test”. The value of Fst was calculated via “Gene Flow and Genetic Differentiation”. The values of Fu’s Fs, FLF and FLD were computed using “Fu and Li’s (and other) Tests”. The different values obtained from COX1, ITS1, and ITS2 gene sequences were recorded.

## 3. Results

### 3.1. Haplotype Analyses

**Figure 1:**
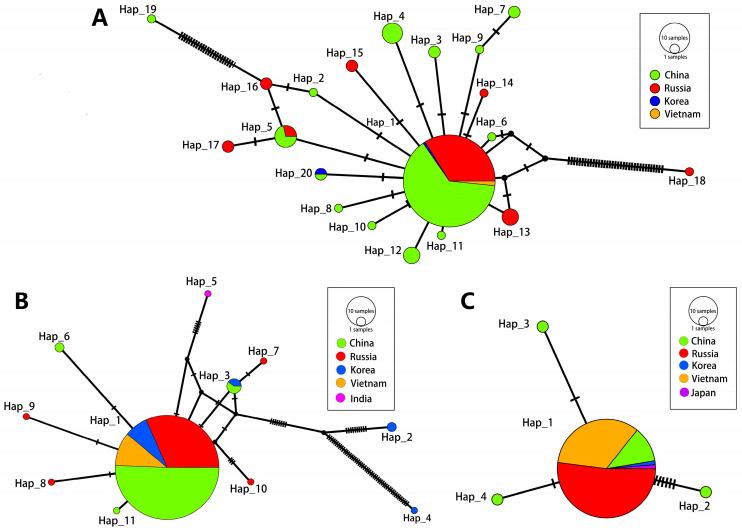
Appearances of *C. sinensis* haplotypes of three sequences. The number of mutations that distinguish haplotypes is indicated by screening marks. The geographical distribution of haplotypes is shown in different colors. The size of the circles is related to haplotype frequency. (**A**): COX1, (**B**): ITS1 and (**C**): ITS2.

There were 20 haplotypes in COX1 (Figure 1A). The largest variety of haplotypes was found in China with 14 species, followed by Russia with 8 species, Korea with 2 species and Vietnam with only 1 species. A total of 11 distinct haplotypes were identified within ITS1 (Figure 1B). The country with the largest number of haplotype species was Russia with five species, followed by China with four species, South Korea with four species, and Vietnam and India with only one species each. There were four haplotypes of ITS2, which were all found in China, while other countries were found to have only Hap 01 (Figure 1C).

As shown in Table S2, the dominant haplotype in 168 COX1 gene sequences was Hap 01, with 125 sequences, accounting for 74.4% (125/168), followed by Hap 05, with 4.17% (7/168). There were eight single haplotypes in the COX1 gene, which came from China (*n* = 6) and Russia (*n* = 2). A total of 11 haplotypes were identified in 275 ITS1 sequences. Among these, Hap 01 was the dominant haplotype, consisting of 259 sequences, accounting for 94.18% (259/275), followed by Hap 03 (1.82%; 5/275). Sequences with one haplotype accounted for 63.64% (7/11), which came from Russia (*n* = 4), Korea (*n* = 1), India (*n* = 1) and China (*n* = 1). Analyses of the ITS2 sequences showed that four haplotypes were recognized. Hap 01 (96.70%; 88/91) was dominant, while three other haplotypes (Hap 02, Hap 03 and Hap 04) were all from a single sequence found in China, accounting for 75% (3/4).

### 3.2. Phylogenetic Analyses

Haplotypes of COX1, ITS1 and ITS2 sequences were used to conduct phylogenetic analyses. Diverse colors were used to represent haplotype distribution in different countries, with green representing China, red representing Russia, blue representing Korea, orange representing Vietnam, purple representing India and gray representing Japan.

**Figure 2:**
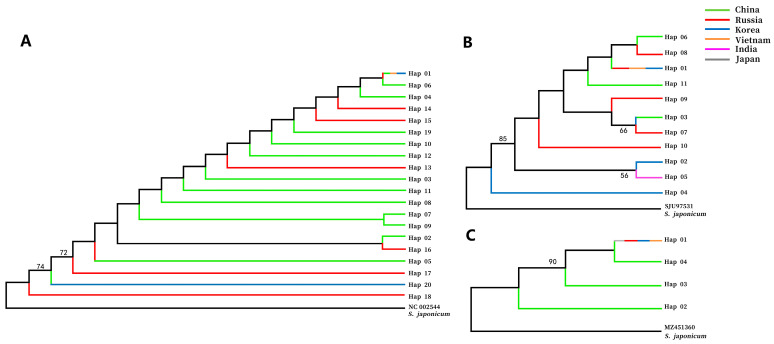
Phylogenetic analysis of *C. sinensis* sequences. (**A**): COX1, (**B**): ITS1 and (**C**): ITS2. Note: (1) The length of the different color line segments only represents the country distribution, and does not represent the proportion of the haplotype to the total haplotype in Figure 2. (2) The bootstrap value section only shows values > 50 in Figure 2.

The relationships between Hap 01 and Hap 06, Hap 07 and Hap 09, and Hap 02 and Hap 16 were closer in the COX1 phylogenetic tree (Figure 2A). Hap 01 was found in all countries. Hap 05 was found in Russia and China, and Hap 20 appeared in China and Korea. Hap 02, Hap 03, Hap 04, Hap 05, Hap 06, Hap 07, Hap 08, Hap 09, Hap 10, Hap 11, Hap 12 and Hap 19 were haplotypes unique to China. Hap 13, Hap 14, Hap 15, Hap 16, Hap 17 and Hap 18 only occurred in Russia.

Hap 06, Hap 08, Hap 01 Hap 11, Hap 09, Hap 03 and Hap 07 clustered together and were closer than others in the ITS1 phylogenetic tree (Figure 2B). Hap 01 was a haplotype that existed in all countries except for India. Hap 03 was found in Korea and China. Hap 06 and Hap 11 were only endemic to China. Hap 07, Hap 08, Hap 09 and Hap 10 were unique haplotypes in Russia. Hap 02 and Hap 04 were haplotypes specific to Korea. Hap 05 only occurred in India.

The relationship between Hap 01 and Hap 04 was the most intimate in the ITS2 phylogenetic tree (Figure 2C). Hap 01 was a shared haplotype for all countries, whereas other haplotypes only existed in China.

### 3.3. Gene Flow, Diversity and Neutrality Analyses

As shown in Table 1, mutations at 56, 83 and 14 different sites were detected in the gene sequences of COX1, ITS1 and ITS2, respectively. Haplotype diversity, Tajima’s D and other relevant values of the COX1, ITS1 and ITS2 groups were calculated separately. The remaining length of the shortened gene sequences was 205 bp for COX1, 241 bp for ITS1 and 250 bp for ITS2. The number of mutations in COX1, ITS1 and ITS2 was 56, 83 and 14, respectively. The value of the parsimony informative sites, the number of haplotypes and Hd were maximum for COX1. The values of π for COX1 and ITS1 were higher than 0.005. To determine whether the population was under selection pressure, Tajima’s D and Fu’s Fs were determined. From the numerical results, Tajima’s D of the ITS1 gene sequences (−2.79729) was lower than that of the COX1 gene sequences (−2.68924) and ITS2 gene sequences (−2.46024), and the differences were extremely significant. The values of Fu’s Fs, FLD and FLF were all lower than zero, and there were extremely significant differences in FLD and FLF. Fst values of the three types of gene sequences were all lower than 0.05.

## 4. Discussion

*C. sinensis* has a deleterious effect not only on economic development and the ecological environment, but also on public safety, particularly in Southeast Asian regions. It is of great importance to conduct epidemiological investigations and molecular-level studies to determine the transmission of this parasite. The findings of this study offer invaluable insights into the genetic evolution and global diversity of *C. sinensis*. The phylogenetic and genetic diversity of a species is indicative of its evolutionary potential and history. Phylogenetic analysis provides insight into the genetic changes that have occurred over time, thus enabling the connection of existing species to their ancestral origins and the prediction of future genetic differentiation [6]. The study of haplotype is of great significance for research on the epidemic distribution and genetic diversity of different parasites. For example, based on the analysis of complete nucleotide sequences of the cob, atp6, nad2, nad1 and cox1 mitochondrial genes in 19 European countries, researchers have suggested that the sequences found in most western, central and eastern European countries, Baltic countries and northeastern Poland might have been inherited from isolated *Echinococcus multilocularis* populations present in ice-covered areas during the glacial period [16]. Alvi et al. detected an extremely high haplotype diversity within both the mt-cox1 and mt-nad1 genes of *Fasciola hepatica* using in silico analysis. The results showed that there were 46 haplotypes for mt-cox1 and 98 for mt-nad1 in 604 samples, and the major haplotypes all shared a common ancestry [17]. Gunyakti et al. analyzed 102 sequences belonging to the mt-CO1 gene of *Taenia multiceps* isolates found in sheep using in silico analysis and identified 20 different haplotypes. The results showed that there were three different haplotype networks (Hap 01, Hap 06 and Hap 12), indicating population development from three main ancestors [5]. In this study, various haplotypes were found in different sequences of *C. sinensis.* The dominant haplotypes isolated from *C. sinensis*, such as Hap 01 in COX1, ITS1 and ITS2, existed in almost all epidemic countries. The three haplotype network analyses showed that haplotypes from the same country were not clustered together. The results of the phylogenetic analyses also indicated that different haplotype sequences from the same country were not in the same group. In addition, there were haplotypes unique to some countries. A hypothesis is that the same haplotype isolated from *C. sinensis* in different regions might have the same ancestor because of animal trade and population movements. The results presented above also supported this hypothesis.

Population genetic diversity plays a crucial role in biodiversity, serving as a reflection of the potential of species to adapt to diverse environmental contexts. The extent, formation mechanism and distribution of genetic diversity can offer insights into the origin and evolutionary trajectory of species, as well as providing information on their potential for evolutionary change [18,19]. Higher levels of genetic diversity have been demonstrated to be associated with greater evolutionary potential and increased responsiveness to environmental changes [20]. Hd and π are important indicators to measure the variation degree of a species population. Gui et al. conducted a genetic diversity analysis of *Dermacentor nuttalli* in Inner Mongolia, China, using Hd and π, and found that it can likely adapt to different geographical environments, thereby leading to genetic diversity and creating genetic differentiation among different populations [21]. In this study, the Hd values of the three types of sequences were all lower than 0.5, indicating low genetic diversity. The reason is related to the fact that this parasite has a deep local adaptation to its environment, including the presence of intermediate hosts and gene flow across the species’ ranges. The values of π for COX1 and ITS1 were both higher than 0.005, indicating high diversity within the population. However, the value of π for ITS2 was lower than the critical value; the reason might be that the number of ITS2 sequences was low, indicating a small population and, thus, a low value of nucleotide diversity. Tatonova et al. identified the existence of inter-individual and intra-genomic variants of ITS1 in *C. sinensis* [22]. The results of this study also showed that there was genetic variation in ITS1. Previous studies examining ITS2 sequences from *C. sinensis* in different countries or from different hosts in the same country showed no significant differences, which was consistent with our results in this study [9,23,24,25,26]. Grant et al. proposed four explanations for the haplotype and nucleotide diversity found in marine fish by combining Hd and π values [27]. According to conjoint analysis of the values found in this study, the low Hd (<0.5) and high π (>0.5) values might be due to divergence between geographically subdivided populations, giving rise to populations with a few highly divergent haplotypes. This condition might have resulted from secondary contact between isolated populations or because of a strong bottleneck in a formerly large, stable population. It was inevitable that formerly isolated marine populations would undergo secondary reassociation [28]. Furthermore, studies on offshore fauna and freshwater organisms have also found low values of Hd (<0.5), while values of π are high (>0.5) [29,30]. It seems reasonable to posit that the intermediate hosts of *C. sinensis*, such as freshwater fish and shrimp, may affect the genetic diversity of its population due to seawater flow and other factors. It is unfortunate that a lack of complete sequence data for intermediate hosts precluded further analysis of their role in the evolution of *C. sinensis* in this study.

In addition to the above analyses on genetic diversity, neutral indices were assessed in this study, supported by a novel hypothesis—the theory of maximum genetic diversity [31]. This theory remains an important component of the exploration of molecular evolution. Neutral indices including Tajima’s D, Fu’s Fs, FLD and FLF were used to assess nucleotide diversity and population expansion [32]. Tajima’s D values for the three types of sequences were all negative in this study, indicating the population of *C. sinensis* was increasing. Tajima’s D for the ITS1 sequence (−2.79729) was lower than that for the COX1 (−2.68924) and ITS2 sequences (−2.46024), indicating that the ITS1 gene sequences had the fastest growth rate. The results were in accordance with the calculation method used by Stephens et al. and Vamathevan et al. [33,34]. A measurement of neutrality is useful for assessing the trends in movement of a species across different countries, and Tajima’s D values in this study suggested that the population growth of *C. sinensis* is likely to accelerate in the future. The values of Fu’s Fs, which is a sign of population growth sensitivity, were significantly negative (*p* < 0.05), indicating that the populations share the same gene pool and have similar growth trends [35,36]. The haplotypes of COX1, ITS1 and ITS2 all had extremely low Fu’s Fs values, indicating that these populations are susceptible to global expansion. Interestingly, the results showed that the values of FLD and FLF were all lower than zero, and the *p*-values showed significant differences. The population of *C. sinensis* might have recently undergone a growing phase and will likely continue with this trend. The results obtained in this study were similar to those obtained in previous studies on *Haemonchus contortus* from seven different geographical locations in China and *Echinococcus ortleppi* in sub-Saharan Africa, Europe and South America; however, not every sequence had Tajima’s D, FLD and FLF values that were significantly different in those studies [37,38]. In light of the aforementioned research findings, it can be concluded that the current population of *C. sinensis* is subjected to less selection pressure and demonstrates an ability to adapt to its surrounding environment. Consequently, the probability of species evolution is relatively low. Fst is a widely accepted and trusted indicator for measuring variability in membership coefficients among individuals in a predefined group, and a value approaching one indicates a high degree of population differentiation [39]. By calculating the Fst value, Gui et al. reported that the genetic differentiation degree of *Dermacentor nuttalli* among some sampled populations was small, while for others, it was moderate [21]. The Fst values of the COX1, ITS1 and ITS2 gene sequences were all lower than 0.05, indicating that genetic differentiation among *C. sinensis* populations was particularly small, likely as a result of high gene flow [40]. The results showed that the mating of *C. sinensis* was random; the genotypes were similar; and geographical isolation was not completely generated. This is beneficial for human public health because it reduces the probability of generating new species. There are several possible reasons for this speculation. First, because China and Russia have more uploaded sequences and obtained more haplotypes than other countries, the differentiation degree of *C. sinensis* populations in the same countries is small. Second, the main endemic countries of *C. sinensis* are close to each other and even contiguous. For example, in China and Russia, there is a large number of suitable hosts for *C. sinensis*, such as *Pseudorasbora parva* in the Wusuli River or the Khanka Lake, which is conducive to the mutual exchange of genes, resulting in a low degree of genetic differentiation. Finally, even if there has been trade between suitable intermediate and terminal hosts of *C. sinensis* between different countries, the genetic similarity of their metacercariae leads to a low degree of adult differentiation. The relatively low Fst value and the limited degree of interspecific differentiation suggest that *C. sinensis* has not undergone positive selection and may have experienced weak negative selection or balancing selection. This finding is in alignment with the results derived from neutral indices.

Unfortunately, although cases of clonorchiasis have been reported in the United States, Canada and Ghana, there are no relevant gene sequences available in GenBank, thus, homology could not be analyzed. The sequences analyzed were mainly from China, Russia and Vietnam, and there were fewer sequences from Korea, Japan and other countries, which might have limited the results regarding the genetic diversity and population growth trend of *C. sinensis*. It is hoped that researchers will upload more sequences, especially NAD1, NAD5 and other valuable gene markers of this liver fluke.

## 5. Conclusions

In conclusion, this is the first study to assess the genetic structure of *C. sinensis* worldwide using in silico analysis. The results indicate that the *C. sinensis* population is growing with less genetic variation, and there are specific variations in *C. sinensis* in different countries. This study provides critical baseline information for future molecular epidemiology and population structure of *C. sinensis* infection.

## Figures and Tables

**Table 1:** Diversity and neutrality indices obtained using nucleotide data of the COX1 (205 bp), ITS1 (241 bp) and ITS2 (250 bp) gene sequences of *C. sinensis*.

Indexes	COX1 (205 bp)	ITS1 (241 bp)	ITS2 (250 bp)
No. of sequences	168	275	91
No. of mutations	56	83	14
Parsimony informative sites	15	2	0
No. of haplotypes	20	11	4
Haplotype diversity (Hd)	0.444 ± 0.049	0.1129 ± 0.026	0.065 ± 0.036
Nucleotide diversity (π)	0.00559 ± 0.00204	0.00768 ± 0.00410	0.00124 ± 0.00104
Tajima’s D	−2.68924 ***	−2.79729 ***	−2.46024 **
Fu’s Fs	−15.056	−4.195	−1.591
FLD	−7.40921 **	−9.53714 **	−6.24115 **
FLF	−6.42975 **	−7.60466 **	−5.57906 **
Fst	0.00994	0.03787	0.00000

Notes: ** means *p* < 0.05; *** means *p* < 0.005.

## Data Availability

The data presented in this study are available on request from the corresponding author.

## Supplementary Materials

The following supporting information can be downloaded at https://www.mdpi.com/article/10.3390/pathogens13110991/s1: Table S1: Accession numbers of COX1, ITS1 and ITS2 gene fragments of *C. sinensis* isolates used in the study; Table S2: Haplotypes of three sequences of *C. sinensis* and numbers of isolates forming each group.

## References

[1] Qian M.B., Zhou X.N. (2021). Clonorchis sinensis. Trends. Parasitol..

[2] Dixon B.R., Flohr R.B. (1997). Fish- and shellfish–borne trematode infections in Canada. Southeast Asian J. Trop. Med..

[3] Fried B., Abruzzi A. (2010). Food-borne trematode infections of humans in the United States of America. Parasitol. Res..

[4] Asare K.K., Boampong J.N., Ameyaw E.O., Thomford A.K., Afoakwah R., Kwakye-Nuako G., Thomford K.P., Quashie N.B. (2014). Microscopic identification of possible *Clonorchis*/*Opisthorchis* infection in two Ghanaian women with undiagnosed abdominal discomfort: Two case reports. J. Med. Case Rep..

[5] Gunyakti Kilinc S., Celik F., Kesik H.K., Simsek S. (2020). In silico analysis of the biodiversity and conservation status of mitochondrial cytochrome *c* oxidase subunit 1 (CO1) gene of *Taenia multiceps*. Acta Parasitol..

[6] Rathnayake R.A.S., Wedage W.M.M., Muthukumarana L.S., De Silva B.G.D.N.K. (2023). Genetic diversity, phylogenetic and phylogeographic analysis of *Anopheles culicifacies* species complex using ITS2 and COI sequences. PLoS ONE.

[7] Dumidae A., Ardpairin J., Pansri S., Homkaew C., Nichitcharoen M., Thanwisai A., Vitta A. (2024). Genetic diversity and population structure of *Physella acuta* (Gastropoda: Physidae) in Thailand using mitochondrial gene markers: COI and 16S rDNA. Sci. Rep..

[8] Chen A.H., Li Z.X., Feng G.N. (2009). Phylogenetic relationships of the genus *Meretrix* (Mollusca: Veneridae) based on mitochondrial COI gene sequences. Zool. Res..

[9] Chelomina G.N., Tatonova Y.V., Hung N.M., Ngo H.D. (2014). Genetic diversity of the Chinese liver fluke *Clonorchis sinensis* from Russia and Vietnam. Int. J. Parasitol..

[10] Solodovnik D.A., Tatonova Y.V., Burkovskaya P.V. (2018). The geographical vector in distribution of genetic diversity for *Clonorchis sinensis*. Parasitol. Res..

[11] Panijpan B., Kowasupat C., Laosinchai P., Ruenwongsa P., Phongdara A., Senapin S., Wanna W., Phiwsaiya K., Kühne J., Fasquel F. (2014). Southeast Asian mouth-brooding Betta fighting fish (Teleostei: Perciformes) species and their phylogenetic relationships based on mitochondrial COI and nuclear ITS1 DNA sequences and analyses. Meta Gene.

[12] Paskewitz S.M., Wesson D.M., Collins F.H. (1993). The internal transcribed spacers of ribosomal DNA in five members of the *Anopheles gambiae* species complex. Insect Mol. Biol..

[13] Lee S.U., Huh S. (2004). Variation of nuclear and mitochondrial DNAs in Korean and Chinese isolates of *Clonorchis sinensis*. Korean J. Parasitol..

[14] Rozas J., Ferrer-Mata A., Sánchez-DelBarrio J.C., Guirao-Rico S., Librado P., Ramos-Onsins S.E., Sánchez-Gracia A. (2017). DnaSP 6: DNA sequence polymorphism analysis of large data sets. Mol. Biol. Evol..

[15] Könnecke M., Akeroyd F.A., Bernstein H.J., Brewster A.S., Campbell S.I., Clausen B., Cottrell S., Hoffmann J.U., Jemian P.R., Männicke D. (2015). The NeXus data format. J. Appl. Crystallogr..

[16] Santoro A., Santolamazza F., Cacciò S.M., La Rosa G., Antolová D., Auer H., Bagrade G., Bandelj P., Basso W., Beck R. (2024). Mitochondrial genetic diversity and phylogenetic relationships of *Echinococcus multilocularis* in Europe. Int. J. Parasitol..

[17] Alvi M.A., Khalid A., Ali R.M.A., Saqib M., Qamar W., Li L., Ahmad B., Fu B.Q., Yan H.B., Jia W.Z. (2023). Genetic variation and population structure of *Fasciola hepatica*: An in silico analysis. Parasitol. Res..

[18] Ellegren H., Galtier N. (2016). Determinants of genetic diversity. Nat. Rev. Genet..

[19] Montero-Pau J., Gómez A., Serra M. (2018). Founder effects drive the genetic structure of passively dispersed aquatic invertebrates. PeerJ.

[20] Hu Y., Fan H., Chen Y., Chang J., Zhan X., Wu H., Zhang B., Wang M., Zhang W., Yang L. (2021). Spatial patterns and conservation of genetic and phylogenetic diversity of wildlife in China. Sci. Adv..

[21] Gui Z., Wu L., Cai H., Mu L., Yu J.F., Fu S.Y., Si X.Y. (2021). Genetic diversity analysis of *Dermacentor nuttalli* within Inner Mongolia, China. Parasite. Vector..

[22] Tatonova Y.V., Chelomina G.N., Besprosvannykh V.V. (2012). Genetic diversity of nuclear ITS1-5.8S-ITS2 rDNA sequence in *Clonorchis sinensis* Cobbold, 1875 (Trematoda: Opisthorchidae) from the Russian Far East. Parasitol. Int..

[23] Park G.M., Yong T.S. (2001). Geographical variation of the liver fluke, *Clonorchis sinensis*, from Korea and China based on the karyotypes, zymodeme and DNA sequences. Southeast Asian J. Trop. Med..

[24] Liu W.Q., Liu J., Zhang J.H., Long X.C., Lei J.H., Li Y.L. (2007). Comparison of ancient and modern *Clonorchis sinensis* based on ITS1 and ITS2 sequences. Acta Trop..

[25] Sun J., Huang Y., Huang H., Liang P., Wang X., Mao Q., Men J., Chen W., Deng C., Zhou C. (2013). Low divergence of *Clonorchis sinensis* in China based on multilocus analysis. PLoS ONE.

[26] Zhang X., Sun B., Tang Q., Chen R., Han S. (2019). Molecular identification and phylogenetic analysis of nuclear rDNA sequences of *Clonorchis sinensis* isolates from human fecal samples in Heilongjiang province, China. Front. Microbiol..

[27] Grant W.A.S., Bowen B.W. (1998). Shallow population histories in deep evolutionary lineages of marine fishes: Insights from sardines and anchovies and lessons for conservation. J. Hered..

[28] Veron J.E.N. (1996). Corals in space and time; the biogeography and evolution of the Scleractinia. Q. Rev. Biol..

[29] Burton R.S. (1986). Evolutionary consequences of restricted gene flow among natural populations of the copepod *Tigriopus californicus*. Bull. Mar. Sci..

[30] Bermingham E., Avise J.C. (1986). Molecular zoogeography of freshwater fishes in the southeastern United States. Genetics.

[31] Huang S. (2016). New thoughts on an old riddle: What determines genetic diversity within and between species?. Genomics.

[32] Ramos-Onsins S.E., Rozas J. (2002). Statistical properties of new neutrality tests against population growth. Mol. Biol. Evol..

[33] Stephens J.C., Schneider J.A., Tanguay D.A., Choi J., Acharya T., Stanley S.E., Jiang R., Messer C.J., Chew A., Han J.H. (2001). Haplotype variation and linkage disequilibrium in 313 human genes. Science.

[34] Vamathevan J.J., Hasan S., Emes R.D., Amrine-Madsen H., Rajagopalan D., Topp S.D., Kumar V., Word M., Simmons M.D., Foord S.M. (2008). The role of positive selection in determining the molecular cause of species differences in disease. BMC Evol. Biol..

[35] Fu Y.X. (1997). Statistical tests of neutrality of mutations against population growth, hitchhiking and background selection. Genetics.

[36] Li Y.L., Kong X.Y., Yu Z.N., Kong J., Ma S., Chen L.M. (2009). Genetic diversity and historical demography of Chinese shrimp *Feneropenaeus chinensis* in Yellow Sea and Bohai Sea based on mitochondrial DNA analysis. Afr. J. Biotechnol..

[37] Yin F., Gasser R.B., Li F., Bao M., Huang W., Zou F., Zhao G., Wang C., Yang X., Zhou Y. (2013). Genetic variability within and among *Haemonchus contortus* isolates from goats and sheep in China. Parasite Vector..

[38] Addy F., Wassermann M., Banda F., Mbaya H., Aschenborn J., Aschenborn O., Koskei P., Umhang G., De La Rue M., Elmahdi I.E. (2017). Genetic polymorphism and population structure of *Echinococcus ortleppi*. Parasitology.

[39] Morrison M.L., Alcala N., Rosenberg N.A. (2022). FSTruct: An F-based tool for measuring ancestry variation in inference of population structure. Mol. Ecol. Resour..

[40] Wright S. (1978). Evolution and the genetics of populations. Variability Within and Among Natural Populations.

