# Supplementary material for: Genetic Variation and Population Structure of Clonorchis sinensis: An In Silico Analysis
Source: Pathogens. 2024 Nov 13;13(11):991. doi: 10.3390/pathogens13110991 (PMC11597292; doi:10.3390/pathogens13110991)
Supplement: Supplementary file 1 [file pathogens-13-00991-s001.zip › Table S1.pdf]

Table S1. Accession numbers of COX1, ITS1and ITS2 gene fragments of *C. sinensis* isolates used in the study.

	COX1		ITS1		ITS2	
**Origin**	**NO.**	**Accession numbers**	**NO.**	**Accession numbers**	**NO.**	**Accession numbers**
Russia	57	MF406175-76-77-78-79-80-81-82-83-84-85-86-87-88-89-90-91-92-93-94-95-96-97-98-99, MF406200-01-02-03-04-05-06, MN116457-58-59-60-61-62-63-64-65-66-67-68-69-70-71-72-73-74-75-76-77-78-79, FJ381664, EU921260	86	JQ048576-77-78-79-80-81-82-83-84-85-86-87-88-89-90-91-92-93-94-95-96-97-98-99, JQ048600-01-02-03-04-05-06-07-08-09-10-11-12-13-14-15-16-17-18-19-20-21, MF319617-18-19-20-21-22-23-24-25-26-27-28-29, KC987514-15-16-17-18-19-20-21-22-23-24-25-26-27-28-29-30-31-32-33-34-35-36-37-38-39, DQ456825	40	EF688143, JQ048576-77-78-79-80-81-82-83-84-85-86-87-88-89-90-91-92-93-94-95-96-97-98-99, JQ048600-01, MF319617-18-19-20-21-22-23-24-25-26-27-28-29
Korea	2	JF729304, KY564177	24	EU038120-21-22-23-24-25-26-27-28-29-30-31-32-33, MW481658-59-60, JN034594-95, JN638318-19-20-21, AF181891	1	JN034597
China	107	JN936207-08-09-10-11-12-13-14-15-16-17-18-19-20-21-22, MT292110-11-12-13-14-15-16-17-18-19-20-21-22-23-24-25-26-27-28-29-30-31-32-33-34-35-36-37-38-39-40-41-42-4	137	MK179278-79-80, KF740423-24-25, KC170164-65-66-67-68-69-70-71-72-73-74-75-76-77-78-79-80-81-82-83-84-85-86-87-88-89-90-91,	23	MK179281-82-83, KF740423-24-25, KU175246, KJ137224-25-26-27-28, PP060703-04-05-06-07-08-09-10-11-12-

		3-44-45-46-47-48-49-50-51-52-53-54-55-56-57-58-59-60-61-62-63-64-65-66-67-68-69-70-71-72-73-74-75-76-77-78-79-80-81-82-83-84-85-86-87-88-89-90-91-92-93-94-97-98-99,<br>MT292200,<br>JF729303,<br>JF739555		AF192414,<br>AF181892,<br>KJ137224-25-26,<br>HQ874523-24-25-26-27-28-29-30-31-32-33-34-35-36-37-38-39-40-41-42-43-44-45-46-47-48-49-50-51-52-53-54-55-56-57-58-59-60-61-62-63-64-65-66-67-68-69-70-71-72-73-74-75-76-77-78-79-80-81-82-83-84-85-86-87-88-89-90-91-92-93-94-95-96-97-98-99,<br>HQ874600-04,<br>HQ186253-60,<br>EU038112-19		13
Vietnam	2	EU652407-08	27	MT497281,<br>MF319630-31-32-33-34-35-36-37-38-39-40-41-42-43-44-45-46-47-48-49-50-51-52-53-54-55	26	MF319630-31-32-33-34-35-36-37-38-39-40-41-42-43-44-45-46-47-48-49-50-51-52-53-54-55
India			1	KT020830		
Japan					1	EF688144
Total		168		275		91
