# Supplementary material for: Genetic Variation and Population Structure of Clonorchis sinensis: An In Silico Analysis
Source: Pathogens. 2024 Nov 13;13(11):991. doi: 10.3390/pathogens13110991 (PMC11597292; doi:10.3390/pathogens13110991)
Supplement: Supplementary file 1 [file pathogens-13-00991-s001.zip › Table S2.pdf]

Table S2. Haplotypes of three sequences of *C. sinensis* and numbers of isolates forming each group.

Sequence name	Haplotype name	NO.	Accession numbers
COX1	Hap 01	125	MT292110-11-China, MT292113-China, MT292115-19-China, MT292122-30-China, MT292133-36-China, MT292138-China, MT292139-China, MT292141-China, MT292145-China, MT292148-50-China, MT292152-55-China, MT292157-62-China, MT292164-China, MT292167-73-China, MT292175-78-China, MT292181-90-China, MT292192-China, MT292194-China, MT292198-99-China, MT292200-China, MN116457-Russia, MN116459-66-Russia, MN116468-72-Russia, MN116475-78-Russia, MF406175-79-Russia, MF406185-Russia, MF406187-99-Russia, MF406200-03-Russia, MF406205-Russia, KY564177-Korea, JN936208-19-China, JN936221-22-China, FJ381664-Russia, EU652407-08-Vietnam
	Hap 02	1	MT292193-China
	Hap 03	2	MT292137-China, MT292191-China
	Hap 04	6	MT292140-China, MT292144-China, MT292147-China, MT292151-China, MT292156-China, MT292197-China
	Hap 05	7	MT292112-China, MT292131-China, MT292174-China, MT292179-China, MF406184-Russia, MF406186-Russia, JN936207-China
	Hap 06	1	MT292180-China
	Hap 07	2	MT292165-66-China
	Hap 08	1	MT292163-China
	Hap 09	1	MT292146-China
	Hap 10	1	MT292143-China
	Hap 11	2	MT292132-China, MT292142-China
	Hap 12	4	MT292114-China, MT292120-21-China, JN936220-China
	Hap 13	4	MN116467-Russia, MF406180-Russia, MF406183-Russia, MF406204-Russia
	Hap 14	1	MN116479-Russia
	Hap 15	2	MN116473-74-Russia
	Hap 16	2	MN116458-Russia, MF406206-Russia

	Hap 17	2	MF406181-82-Russia
	Hap 18	1	EU921260-Russia
	Hap 19	1	JF739555-China
	Hap 20	2	JF729303-China, JF729304-Korea
ITS1	Hap 01	259	MF319617-29-Russia, JN638318-Korea, JN638320-Korea, JN034594-95-Korea, EU038131-Korea, MW481658-60-Korea, EU038112-19-China, EU038120-30-Korea, AF192414-China, AF181892-China, HQ874523-99-China, HQ874601-04-China, HQ186254-China, HQ186257-China, HQ186259-60-China, KC987514-39-Russia, JQ048576-99-Russia, JQ048600-03-Russia, JQ048605-06-Russia, JQ048608-19-Russia, JQ048621-Russia, KF740423-25-China, KC170164-91-China, KJ137226-China, MK179278-80-China, MF319630-55-Vietnam, MT497281-Vietnam
	Hap 02	2	JN638319-Korea, JN638321-Korea
	Hap 03	5	HQ186253-China, HQ186256-China, EU038132-33-Korea, KJ137225-China
	Hap 04	1	AF181891-Korea
	Hap 05	1	KT020830-India
	Hap 06	2	HQ186255-China, HQ186258-China
	Hap 07	1	JQ048607-Russia
	Hap 08	1	JQ048620-Russia
	Hap 09	1	JQ048604-Russia
	Hap 10	1	DQ456825-Russia
	Hap 11	1	KJ137224-China
ITS2	Hap 01	88	EF688143-Russia, EF688144-Japan, JN034597-Korea, JQ048576-99-Russia, JQ048600-01-Russia, KF740423-24-China, KJ137226-28-China, KU175246-China, MF319617-29-China, MK179281-83-China, MF319630-55-Vietnam, PP060703-13-China
	Hap 02	1	KF740425-China
	Hap 03	1	KJ137224-China
	Hap 04	1	KJ137225-China
